# Comparison of cold crystalloid and colloid infusions for induction of therapeutic hypothermia

**DOI:** 10.1186/cc10889

**Published:** 2012-03-20

**Authors:** RS Skulec, AT Truhlar, ZT Turek, RP Parizkova, VC Cerny

**Affiliations:** 1 Charles University in Prague, University Hospital Hradec Kralove, Czech Republic

## Introduction

While cold crystalloids have been used for induction of therapeutic hypothermia after cardiac arrest [1,2], the effectiveness of cold colloids has not so far been evaluated. Therefore, we investigated the cooling effect of rapid intravenous infusion of cold crystalloid compared to colloid in a porcine model of ventricular fibrillation (VF).

## Methods

VF was electrically induced in 22 anesthetized domestic pigs (33 ± 2 kg). Defibrillation was attempted after 15 minutes CPR using the AutoPulse (Zoll Medical, USA) and artificial ventilation. After spontaneous circulation was restored, the animals were randomized to receive either 1,500 ml of 1°C cold normal saline (group A; *n *= 9) within 20 minutes using a Zoll Power Infuser, or 1,500 ml of 1°C cold Voluven (6% hydroxyethyl starch 130/0.4 in 0.9% NaCl) (group B; *n *= 9), or no infusion (group C; *n *= 4). The animals were observed for 90 minutes following infusion. Cerebral, rectal, intramuscular, pulmonary artery, and subcutaneous fat body temperatures (BT) were continuously recorded using GES 130 temperature probes and GMH 3250 digital thermometers (Greisinger Electronic, Germany). Data were analyzed with JMP 3.2 software (SAS Institute, USA) and are expressed as a mean ± SD. *P *< 0.05 was considered statistically significant.

## Results

In total, 46.6 ± 3.2 ml/kg cold normal saline was infused in group A, and 45.7 ± 2.7 ml/kg cold colloid in group B. The animals treated with cold fluids achieved a significant decrease of BT in all measurement sites while there was a spontaneous increase in group C (*P *< 0.05). At the time of finishing infusion there was a greater decrease in cerebral and pulmonary artery BT in group A compared to group B (-1.7 ± 0.4 vs. -1.1 ± 0.3 °C, *P *= 0.002; and -2.1 ± 0.3 vs. -1.6 ± 0.2 °C, *P *< 0.001 respectively). Area under the curve analysis of the decrease in intracerebral BT revealed a more vigorous cooling effect in group A compared to B (-91 ± 30 vs. -62 ± 27 °C/minute, *P *= 0.047). There was also a higher calculated enthalpy for crystalloid solution compared to colloid in the time-point of maximal BT decrease (33.9 ± 5.7 vs. 26.6 ± 3.4 kJ/kg, *P *< 0.05).

## Conclusion

Cold crystalloid infusions resulted in a more intense cooling effect than colloid infusions of the same temperature and infusion rate in this porcine model of cardiac arrest.
